# Network pharmacology analysis and clinical verification of Jishe Qushi capsules in rheumatoid arthritis treatment

**DOI:** 10.1097/MD.0000000000034883

**Published:** 2023-08-25

**Authors:** Yujie Li, Nannan Zhang, Xin Peng, Wukai Ma, Yuanxing Qin, Xueming Yao, Cong Huang, Xudong Zhang

**Affiliations:** a The Second Affiliated Hospital of Guizhou University of Traditional Chinese Medicine, Guiyang, P. R. China; b School of Basic Medicine, Guizhou University of Traditional Chinese Medicine, Guian District, Guiyang, P. R. China; c Guizhou Province Key Laboratory of Prescription and Syndrome Pharmacology in Chinese Medicine, Guian District, Guiyang, P. R. China; d College of Acunox and Tuina, Guizhou University of Traditional Chinese Medicine, Guian District, Guiyang, P. R. China.

**Keywords:** clinical verification, Jishe Qushi capsules, network pharmacology, rheumatoid arthritis

## Abstract

The study aimed to elucidate the effective chemical composition and molecular mechanism of rheumatoid arthritis (RA) treatment with Jishe Qushi capsules (JSQS) and perform clinical validation. The effective chemical components were screened by a database. We used Cytoscape software to construct the key target-RA composite target network of JSQS. Gene Ontology biofunctional analysis and Kyoto encyclopedia of Genes and Genomes (KEGG) pathway enrichment analysis were performed for the key targets, followed by molecular docking validation of core key targets. Ninety-nine patients chosen were divided into 49 cases in the treatment group and 50 cases in the control group according to the random number table method. The control group was treated with the combination of methotrexate (MTX) plus Glucosidorum Tripterygll Totorum. The treatment group was treated with MTX plus JSQS. The treatment effects of the 2 groups were evaluated. A total of 118 key anti-RA targets were obtained for JSQS. Quercetin in *Panax notoginseng*, vanillic acid, scopoletin, physcion in *Gardneria angustifolia*, 3,5-dimethyl-4-hydroxybenzaldehyde in *Zaocys dhumnades*, kaempferol in *Radix Paeoniae Alba*, and protocatechuic aldehyde in *Cibotium barometz* were the main active chemical components in the composite target network. Topology analysis yields core key targets, such as TP53, INS, IL6, VEGFA, MYC, CASP3, ESR1, EGF, CCND1, PPARG, ERBB2, NFKBIA, TLR4, RELA, and CASP8, and the results of KEGG enrichment analysis showed that JSQS mainly works through pathways in cancer, phosphatidylinositol-3-kinaseRAC–serine/threonine-protein kinase signaling, and mitogen-activated protein kinase (MAPK) signaling pathway, and aryl hydrocarbon receptor nuclear translocator signaling pathway. Molecular docking results showed that the binding fraction of PPARG, VEGFA and the effective active ingredients of ridged snake dispelling capsule was >70. In the clinical trial, morning stiffness, joint pain, and VAS scores of post-treatment in the treatment group were lower than those in the control group (*P* < .05). Additionally, ESR, CRP, RF, anti-CCP, TNF-α, IL-6, IL-17, and Th17/Treg were lower in the treatment group than in the control group (*P* < .05). JSQS exert multicomponent, multipathway, and multitarget synergistic actions in RA treatment. It can significantly improve the clinical symptoms and quality of life and delay the progression of RA disease.

## 1. Introduction

Rheumatoid arthritis (RA) is a common immune disease characterized by the symmetrical destruction of multiple joints.^[1]^ The prevalence of RA in China is 0.32% to 0.36%,^[2]^ and RA incidence is on the rise.^[3]^ If not thoroughly treated, RA gradually leads to cartilage erosion and, ultimately, joint deformity, causing great pain to patients and seriously affecting their quality of life. Thus, RA has gradually become one of the serious diseases affecting people health. RA is still the number one joint disease causing disability in China^[4]^; hence, RA prevention and treatment have been an important research topic.^[5]^ Nevertheless, the specific pathogenesis of RA is not fully clarified.^[6]^ At this stage, there is still a lack of ideal drugs and methods for RA treatment. The first-line antirheumatic agents, such as methotrexate (MTX), iguratimod, and leflunomide, can delay RA progression but still lack ideal effects on the progression of bone destruction. Moreover, biologics and targeted drugs are more expensive and cause certain serious adverse effects; therefore, most patients cannot adhere to them.

Chinese traditional medicine has a curative effect in the treatment of RA. These medicines—such as Glucosidorum Tripterygll Totorum and total glucosides of *Paeonia*, which are modern drugs developed based on the traditional theory of Chinese medicine—have definite efficacy for patients who cannot tolerate immunosuppressants or the poor effect of biologics and are very important and effective complementary alternative medical treatments. Jishe Qushi capsules (JSQS) (Qian Medicine System Z20160028) were developed by the Miao medicine Jin Wu Jian Gu founded by Professor WuKai Ma of the Department of Rheumatology and Immunology of the Second Affiliated Hospital of Guizhou University of Traditional Chinese Medicine. The results of a randomized controlled trial^[7]^ have shown that JSQS effectively relieved and improved the symptoms of RA and slowed down disease progression, with definite clinical efficacy. Additionally, the annual clinical reception of related patients was more than 50,000, with the annual sales of the in-hospital preparation of JSQS exceeding 5 million yuan.^[8,9]^ However, the drug is an empirical formula, and laboratory studies are lacking. This study used a combination of network pharmacology and clinical control trials to elucidate the material basis and molecular mechanism of JSQS in RA treatment.

## 2. Materials and methods

### 2.1. Network pharmacology

#### 2.1.1. Acquisition and screening of the chemical composition of JSQS.

The Traditional Chinese Medicine Systems Pharmacology Database and Analysis Platform (TCMSP) database was used to retrieve all chemical components of the Chinese herbal medicine composition of *Paeonia lactiflora, Panax notoginseng, Homalomena occulta, Curcuma longa*, and *Caulis Sinomenii* for the collection of active ingredients and protein targets using oral bioavailability of ≥30% and drug similarity (DL) of ≥0.18 as screening conditions. Because the TCMSP database did not provide the chemical components of *Zaocys dhumnades, Cibotium barometz*, and *Gardneria angustifolia*, we searched for them in the literature, and the protein targets of the compounds were collected using the ChemMapper database. After the targets retrieved from both data platforms were merged and de-duplicated, the protein target names were normalized using the Uniprot protein database, setting human as the species parameter.

#### 2.1.2. Collection of disease target information and screening of cross-targets.

Using “rheumatoid arthritis” as the keyword, we searched the GeneCards and Online Mendelian Inheritance in Man human gene platforms for confirmed genes related to RA, downloaded the database into Excel, and de-duplicated the genes. The cross-targets of diseases and drugs were obtained by using Weishengxin.

#### 2.1.3. Construction of protein interactions.

The targets of RA intersection with JSQS were imported into the String database, and the species was set as “homo sapiens.” Additionally, the minimum interaction score correlation was set as “highest confidence” (≥0.400). The drug–disease protein–protein interaction (PPI) network map was initially obtained by downloading the TSV file and processing it with Cytoscape 3.90 software. The core targets were selected by analyzing the network degree and other parameters with the help of the plug-in Network Analyzer.

#### 2.1.4. Construction of network model.

The compounds and related targets obtained from JSQS were categorized, as well as the targets that intersected with the diseases. The active ingredient-target network of the capsules for RA treatment was constructed by Cytoscape 3.90 software. The network topology parameters, such as degree and betweenness, were used to screen the key active ingredients of the drug using the built-in tools of the software.

#### 2.1.5. GO enrichment analysis and KEGG pathway enrichment analysis.

The drug-disease intersection targets were imported into the Database for Annotation, Visualization and Integrated Discovery (DAVID), and the species was set as human species. The kyoto encyclopedia of genes and genomes (KEGG) pathway and Gene Ontology (GO) enrichment analyses were submitted to establish a “core target-biological pathway” network to screen out the major pathways. The KEGG enrichment bubble chart and GO term enrichment were created online using the results of bioinformatics analysis to reflect the interactions between drug targets and RA target-related pathways.

### 2.2. Analysis of molecular docking technology

We obtained the 3D crystal structure of the target protein from the RCSB PDB (http://www.rcsb.org/) protein crystal structure database or the Uniprot database. Then, we obtained the 3D structure of the compound corresponding to the protein we wanted to analyze from the TCMSP database, followed by importing the protein and compound into Discovery Studio 2016 Client software for molecular docking.

### 2.3. Clinical controlled trials

#### 2.3.1. General clinical information

Ninety-nine patients with RA treated at the Second Affiliated Hospital of Guizhou University of Traditional Chinese Medicine from January 2021 to 2022 were selected for the study. Among them, 18 cases were men, and 81 cases were women. Their age ranged from 24 to 75 years, with an average age of 56.34 ± 12.80 years. Disease duration ranged from 7 months to 11 years, with an average disease duration of 5.21 ± 2.74 years. Inclusion criteria comprised: patients aged 18 to 60 years; patients that met the diagnostic criteria of active RA according to Chinese Guidelines for Rheumatoid Arthritis Treatment (2018 edition); patients diagnosed for the first time, patients who did not receive formal drug treatment after diagnosis, or patients who received formal treatment but stopped taking drugs for more than 3 months; and patients with 2.6 ≤ Disease Activity Score using 28 joint counts <5.1, joint function classification of I to III, and X-ray stage I to III of both hands.

Exclusion criteria were: patients aged <18 years or >60 years; patients with grade IV joint function and/or X-ray stage IV of both hands; patients with a history of hormone and/or biological treatment within 1 month; patients with severe cardiac, hepatic, renal, or another important organ insufficiency; patients with other serious diseases, such as malignancy, active gastrointestinal ulcer, and infectious diseases; patients with severe extra-articular manifestations, such as peripheral neuropathy, proteinuria, and interstitial pulmonary fibrosis; and patients with allergy to the drug of our study.

#### 2.3.2. Drug

The control group received MTX (16 tablets/bottle, Shanghai Xinyi Pharmaceutical Co., Ltd., Lot no. 036180503) 10 mg once a week and Glucosidorum Tripterygll Totorum (100 tablets/bottle, Guizhou Hanfang Pharmaceutical Co., Ltd., lot no. 1459001) 20 mg thrice daily for 4 consecutive weeks. The treatment group received MTX, 10 mg once a week, combined with JSQS (0.45 g, 36 capsules/bottle, from Department of Pharmacy, Second Affiliated Hospital of Guizhou University of Traditional Chinese Medicine, lot no. 20180101) 4 capsules each time, thrice daily for 4 consecutive weeks. Both groups took folic acid tablets 10 mg (100 tablets/bottle, Changzhou Pharmaceutical Factory Co., Ltd., lot no. 18082511) on the next day during the MTX administration. Patients of both groups were allowed to take celecoxib capsules (6 capsules/box, Pfizer Pharmaceutical Co., Ltd., lot no. W64713) orally at a maximum dose of 2 capsules per day during the study period due to joint pain, and the number of taken capsules and the withdrawal time were recorded separately for both groups.

The criteria for determining efficacy were formulated with reference to the Guiding Principles for Clinical Research on New Chinese Medicines (Trial) 2020. Efficacy was defined as the overall improvement rate of patients’ main symptoms and signs of ≥75%, while erythrocyte sedimentation rate (ESR) and C-reactive protein (CRP) were normal or significantly improved. Progress was defined as the overall improvement rate of patients’ main symptoms and signs of ≥50%, while ESR and CRP improved. Effectiveness was defined as the overall improvement rate of patients’ major symptoms and signs of ≥30%, while ESR and CRP were improved or not improved. Ineffectiveness was defined as the overall improvement rate of patients’ main symptoms and signs of <30%, while ESR and CRP did not improve. The total effective rate was calculated as follows:

Total effective rate = (significant + improvement + effective)/ total number of cases.

#### 2.3.3. Observed indicators.

##### 2.3.3.1. Comparing the main symptoms and signs of the 2 groups.

The joint pressure pain, swollen joint count, time of morning stiffness, and visual analog scale (VAS) of the 2 groups were compared before and after treatment. As VAS, a 10-cm scale was selected, with 0 representing a completely pain-free state and 10 representing intolerably severe pain. Patients chose the corresponding scale according to their pain level, and the physician in charge chose the corresponding score.

##### 2.3.3.2. Comparing the level of changes in key clinical indicators.

First, 5 mL of fasting venous blood was collected from patients in both groups before and after treatment. After centrifugation at 3000 r/min for 10 minutes, the supernatant was pretreated and stored at low temperature and used to detect the relevant indexes of patients. Blood ESR was measured by the Westergren methods. CRP was measured by immunodiffusion. Rheumatoid factor (RF) was measured by latex agglutination test. Anti-cyclic citrullinated peptide (anti-CCP) was measured by enzyme-linked immunosorbent assay. The reagents were purchased from Shanghai Chemical Biotechnology Co., and the operation procedure was performed strictly according to the manufacturer instructions.

##### 2.3.3.3. Comparing the levels of inflammatory factor changes in the 2 groups.

Five mL of fasting venous blood was collected from patients in both groups before and after treatment. After centrifugation at 3000 r/min for 10 minutes, the supernatant was pretreated and stored at 4°C and used to detect the relevant indexes of patients. The levels of serum interleukin-6 (IL-6), serum interleukin-17 (IL-17), and tumor necrosis factor (TNF-α) in the 2 groups were measured by enzyme-linked immunosorbent assay. The respective kits were purchased from Shanghai Enzyme Link Biotechnology Co., and the operation procedure was performed strictly according to the manufacturer instructions.

##### 2.3.3.4. Comparing the level of changes in immune function indexes in the 2 groups.

First, 5 mL of fasting venous blood was collected from patients in both groups before and after treatment. After centrifugation at 3000 r/min for 10 minutes, the supernatant was taken for pre-treatment. The changes in the expression levels of helper T cells 17 (Th17) and regulatory T cells (Treg) were detected using a flow cytometer (Serena [China] Medical Technology Co., Ltd, model Sparrow), and the operation procedure was performed strictly according to the manufacturer instructions.

### 2.4. Observation of adverse reactions

We observed the adverse reactions during treatment in both groups. No obvious clinical signs and symptoms of discomfort were found.

### 2.5. Statistical methods

Statistical differences were considered as significant if the *P* value was below .05. All analyses were performed using IBM SPSS Statistics, version 23.0 (IBM SPSS Inc., Chicago). The *t* test was used for measurement data, expressed as ‾*x ± *s. The χ^2^ test was used for count data.

## 3. Results

### 3.1. Acquisition and screening of the chemical composition of Chinese herbal medicines in JSQS

A total of 33 active compounds were collected from *Paeonia lactiflora, Panax notoginseng, Homalomena occulta, Curcuma longa*, and *Caulis Sinomenii* through the TCMSPS platform with settings of oral bioavailability of ≥30% and DL of ≥0.18, as shown in Table 1. Sixty-six potentially active compounds were retrieved from 3 Chinese herbal medicines—namely *Zaocys dhumnades, Cibotium barometz* and *Gardneria angustifolia*—in ChemMapper (71 potential compounds, of which 5 compounds were excluded), as shown in Table 2.

**Table 1:** Potential active ingredients of JSQS (TCMSP).

Chinese herb	Mol ID	Molecule name	OB	DL
	MOL001910	11alpha,12alpha-epoxy-3beta- 23-dihydroxy- 30-norolean-20-en-28,12betaolide	64.77	0.38
	MOL001918	paeoniflorgenone	87.59	0.37
	MOL001919	(3S,5R,8R,9R,10S,14S)-3,17- dihydroxy-4,4,8,10,14- pentamethyl-2,3,5,6,7,9- hexahydro-1H-cyclopenta[a]phenanthrene- 15,16-dione	43.56	0.53
	MOL001921	lactiflorin	49.12	0.8
	MOL001924	paeoniflorin	53.87	0.79
	MOL001925	paeoniflorin_qt	68.18	0.4
	MOL001928	albiflorin_qt	66.64	0.33
	MOL001930	benzoyl paeoniflorin	31.27	0.75
	MOL000211	mairin	55.38	0.78
	MOL000358	beta-sitosterol	36.91	0.75
	MOL000359	sitosterol	36.91	0.75
	MOL000422	kaempferol	41.88	0.24
*Radix Paeoniae Alba*	MOL000492	catechin	54.83	0.24
	MOL000449	stigmasterol	43.83	0.76
	MOL000493	campesterol	37.58	0.71
*Rhizoma Curcumae longae*	MOL000953	CLR	37.87	0.68
	MOL000358	beta-sitosterol	36.91	0.75
	MOL000686	acetylbullatantriol	40.21	0.18
*Rhizoma Homalomenae*	MOL000691	maristeminol	30.64	0.38
	MOL000358	beta-sitosterol	36.91	0.75
	MOL000621	16-epi-Isositsirikine	49.52	0.59
	MOL000622	magnograndiolide	63.71	0.19
	MOL000623	michelenolide	47.54	0.25
	MOL000625	sinomenine	46.09	0.53
*Caulis Sinomenii*	MOL000627	stepholidine	33.11	0.54
	MOL001494	mandenol	42	0.19
	MOL001792	DFV	32.76	0.18
	MOL002879	diop	43.59	0.39
	MOL000358	beta-sitosterol	36.91	0.75
	MOL000449	stigmasterol	43.83	0.76
	MOL005344	ginsenoside rh2	36.32	0.56
	MOL007475	ginsenoside f2	36.43	0.25
*Radix Notoginseng*	MOL000098	quercetin	46.43	0.28

CLR = Cholesterol, DFV = 4',7-Dihydroxyflavanone, DL = drug similarity, OB = oral bioavailability.

**Table 2:** Potential active ingredients of JSQS (ChemMapper).

Chinese herb	Mol ID	Molecule name	Mol ID	Molecule name
	MOL001973	β-sitosterol	MOL001988	stearic acid
	MOL001974	hexadecanoic acid	MOL001989	diethyl phthalate
	MOL001975	5-hydroxymethyl furaldehyde	MOL001990	tetradecanoic acid
	MOL001976	cirsiumaldehyde	MOL001991	linoleic acid ethyl ester
	MOL001977	daucosterol	MOL001992	methyl palmitate
	MOL001978	3-hydroxyl-γ-pyrone	MOL001993	ethyl stearate
	MOL001979	γ-terpinen	MOL001993	p-hydroxyacetanilide
	MOL001980	piperitone	MOL001995	protocatechuic aldehyde
	MOL001981	cadina-1(10)4-diene	MOL001996	vanillin
	MOL001982	7,10,13-hexadecatrienoic acid methyl ester	MOL001998	(24R)-stigmnst-4-ene-3-one
	MOL001983	3-terpineol	MOL001999	24-methylenecyeloartanol
	MOL001984	eicosatetraenoate methyl	MOL002000	lasiodiplodin
	MOL001989	dibutyl phthalate	MOL002001	onitin
	MOL001985	-9-octadecenoic acid	MOL002003	glucopyranoside
	MOL001986	-9-octadecenoic acid	MOL002003	glucopyranoside
	MOL001987	linolenic acid methyl ester	MOL002002	altemariol
*Cibotium barometz*	MOL001984	(z, z, z)-9, 12, 15-octadecatrien-1-o1		
	MOL001374	Δ5-pregnene-3β,17α,20α-triol	MOL001061	3-O-caffeoylquinic acid methyl ester
	MOL000016	27-hydroxyl-α-amyrin	MOL001233	syringic acid
	MOL000031	ursolic acid	MOL001245	vanillic acid
	MOL000033	β-sitosterol	MOL000001	α-amyrin
	MOL000047	daucosterol	MOL001402	chrysophanol
	MOL000061	emodin	MOL001608	tanshinone IIA
	MOL000225	physcion	MOL001685	soy isoflavone
	MOL000241	phenacetin	MOL001888	liquiritigenin
	MOL000274	hexadecanoic acid	MOL001916	linalool
	MOL000404	3β-hydroxy-oleana-ll, 13(18)-dien-28-oic acid	MOL001075	1,3-di-O –caffeoylquinic acid
	MOL000407	14-ursen-3-ol-1-one	MOL001816	formononetin
	MOL000433	kaempferol	MOL001089	protocatechuic aldehyde
	MOL000481	quercetin	MOL001943	anethole
	MOL000536	scopoletin	MOL000936	n-heptadecane
	MOL000522	emodin-8-O-β-Dglucopyranoside	MOL000508	glucopyranoside
	MOL000666	E-p-hydroxy-cinnamic acid	MOL000867	Caffeic acid
*Periploca Forrestii Schltr*.	MOL000498	kaempferol-3-O-β-Dgalactopyranoside		
	MOL000017	ihydroferulic acid	MOL000145	thymine
	MOL000131	β-sitosterol	MOL000283	4-hydroxybenzaldehyde
*Zaocys*	MOL000001	utylisobutyl phthalate		

### 3.2. Collection of disease target information and screening of cross-targets

**Figure 1.**
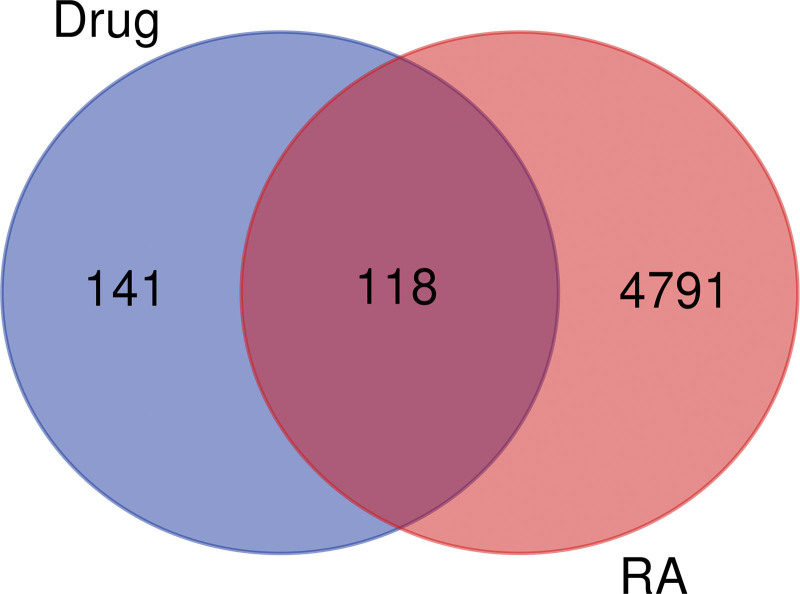
Cross-targeting of disease and drug. Note: pink represents the number of targets for RA, blue represents the number of targets for active constituents of Jishe Qushi capsules, and the middle part represents the intersection targets of the 2. RA = rheumatoid arthritis.

A total of 4881 and 28 RA-related targets were obtained from the GeneCards and Online Mendelian Inheritance in Man databases, respectively. The RA targets collected from the 2 databases were integrated and de-duplicated, providing a total of 4791 targets. The disease and drug targets were imported into Weishengxin to obtain the Venn diagram, as shown in Figure 1. There were 118 cross-targets of RA with JSQS, which are shown in Table 3.

**Table 3:** Common targets of Jishe Qushi capsules in RA.

Name	Number	Target name
Common targets	118	CCNB1 VKORC1 BCL2 MAPK10 GSTP1 AHR NFE2L2 HDAC3 CASP1 SCNN1B VEGFA MYC GSK3B RHO TRPV1 PROC MMP3 ERBB2 CEACAM5 F10 RUNX2 SEC14L2 TYR ACP1 PIK3R1 CHRM3 NFKBIA SDHA CHRNA7 CHRNE IRF1 ACACA PON1 CS SELE SLC25A13 ACHE SHMT1 IL6 CASP3 COL3A1 P4HA2 SEPSECS NQO1 ERBB3 CAV1 HNF4A RELA TLR4 JAK1 SLC25A12 DLAT AR GAD1 CDH5 CYP3A4 SCN9A HBA2 HSPB1 DCAF5 BIRC5 CRP NR1I3 HCK EGF SPP1 GAD2 CCND1 ESR1 PLOD2 STAT1 HMOX1 INS GSTM1 PLAT IKBKB TLR7 SRD5A1 MAPK8 PIK3R2 RB1 NFS1 KRT7 TP53 CASP9 CHRNG APRT NGF LBP CASP8 ARF1 PPARG RAF1 FURIN FGFR2 ATP5F1A PPIA CTSD PARP1 PDE2A CHRNA1 DNMT1 CSAD ICAM1 HDAC2 CA2 DDC HSF1 CHRM2 OPRM1 VCAM1 SCNN1A CFB MIF PTGS1 PRKCA HDAC6 TOP1

### 3.3. Construction of protein interactions

**Figure 2.**
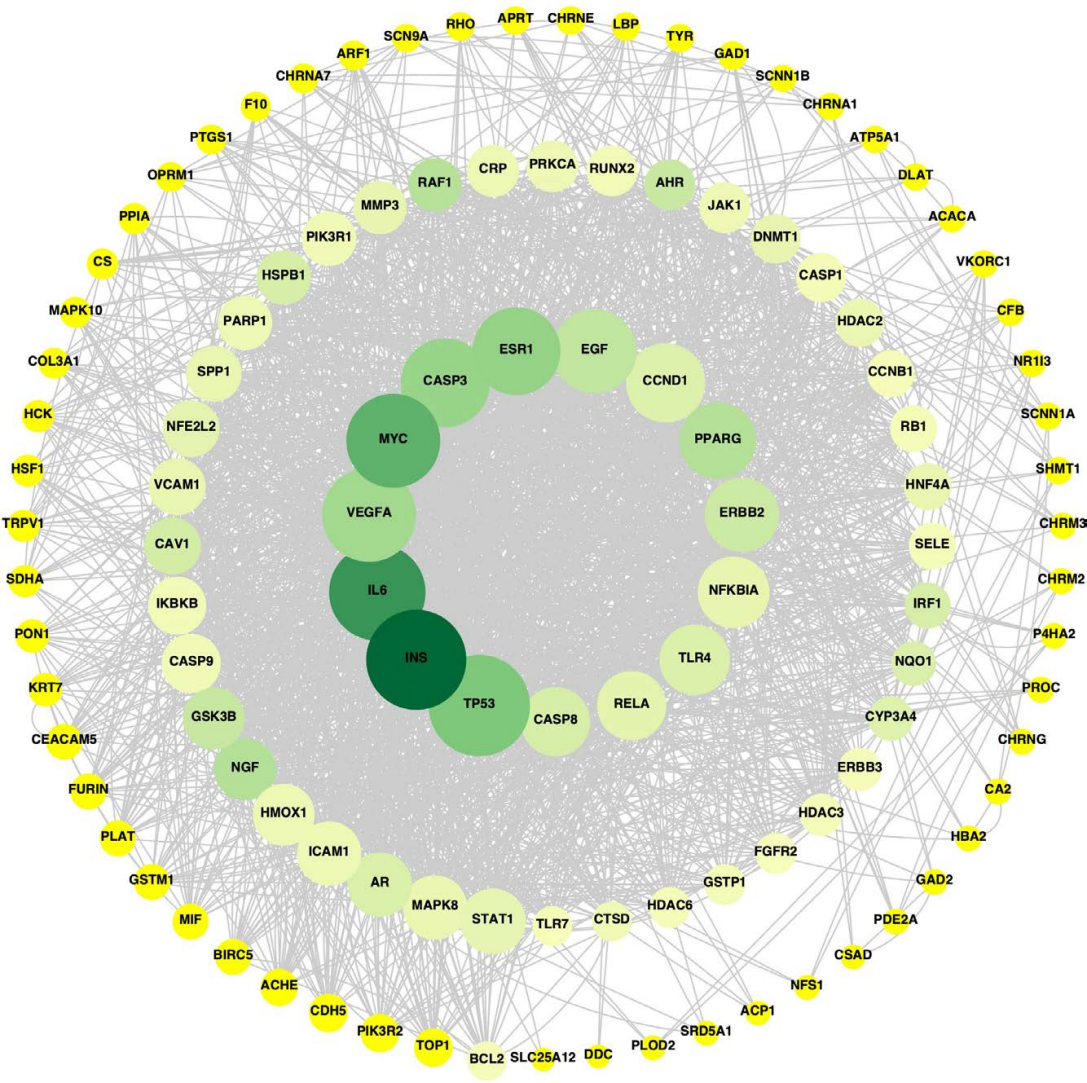
Jishe Qushi capsules and RA have a common target PPI network. The circle represents the disease target, and the line represents the relationship between the targets. PPI = protein–protein interaction, RA = rheumatoid arthritis.

A total of 118 targets based on the intersection of JSQS and RA were imported into the String platform. The PPI network map was initially obtained after setting the corresponding parameters, and the downloaded TSV file was opened in Cytoscape 3.90 software to draw the PPI network map. The larger the value of Degree and Betweenness Centrality, the larger the shape of the node, and the larger the value of Betweenness Centrality, the closer the color of the node was to dark green, as shown in Figure 2. The core targets were TP53, INS, IL6, VEGFA, MYC, CASP3, ESR1, EGF, CCND1, PPARG, ERBB2, NFKBIA, TLR4, RELA, and CASP8.

### 3.4. Compound-intersection targets-disease network of JSQS

**Figure 3.**
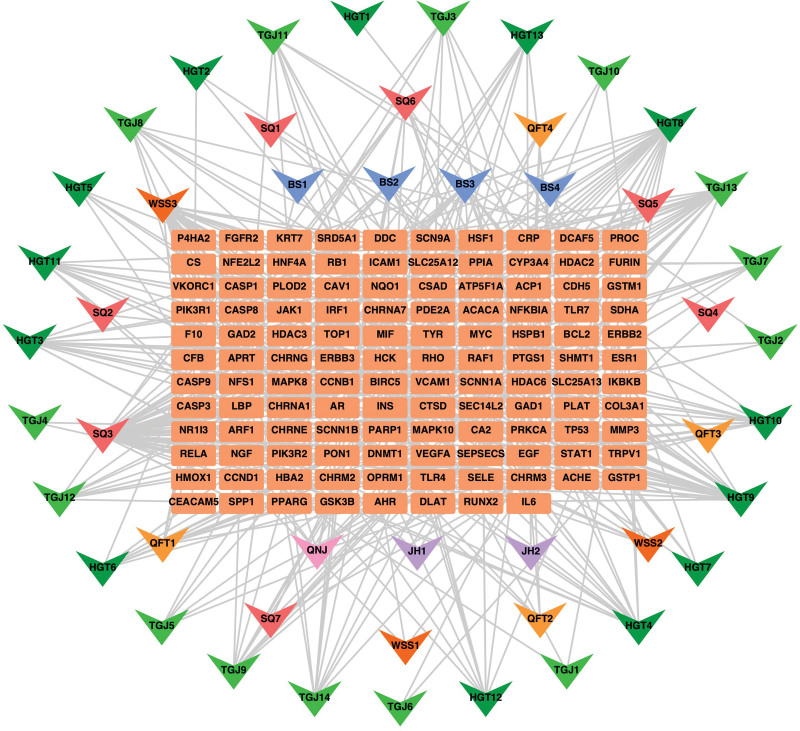
The orange rectangle represents the target point where the JSQS and RA intersect, and the other triangular nodes represent the active ingredients of the drug, with the same color attributed to the same herbal medicine. JSQS = Jishe Qushi capsules, RA = rheumatoid arthritis.

One hundred eighteen common targets were used to construct a “Chinese herbal medicine-active ingredient-target” network containing 166 nodes and 423 edges, using Cytoscape software, as shown in Figure 3. The degree of each node was calculated by the built-in data analysis, and the higher the degree value, the higher the possibility of the compound playing a therapeutic role. Seven key potent molecules were screened by degree values in the network, namely quercetin, vanillic acid, 4-hydroxybenzaldehyde, kaempferol, protocatechuic aldehyde, scopoletin, and physcion, as demonstrated in Table 4.

**Table 4:**
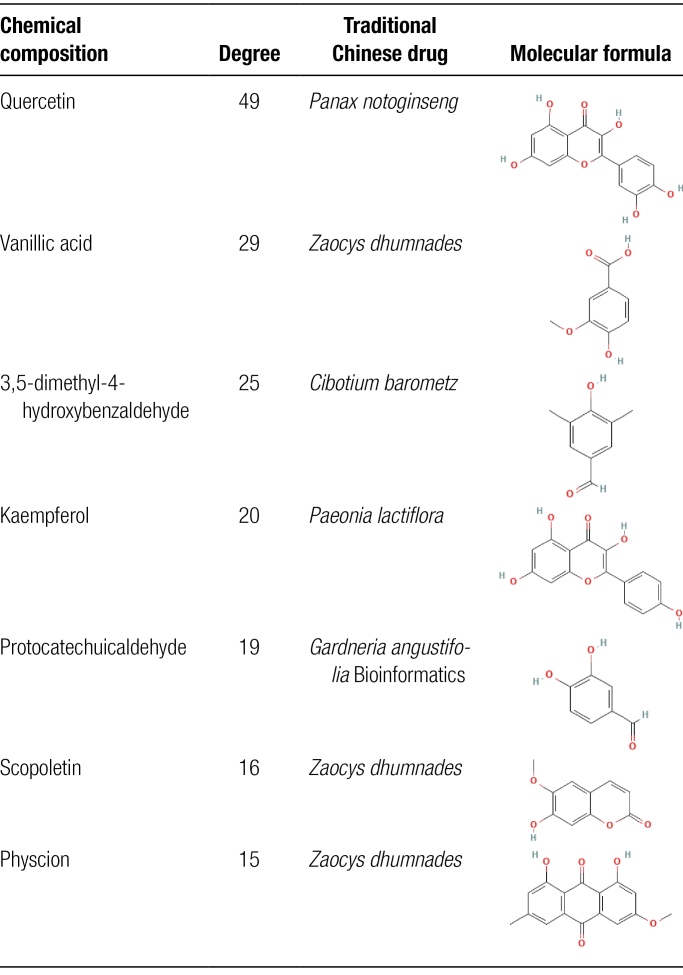
Basic information on key compounds.

### 3.5. GO enrichment analysis and KEGG pathway enrichment analysis

**Figure 4.**
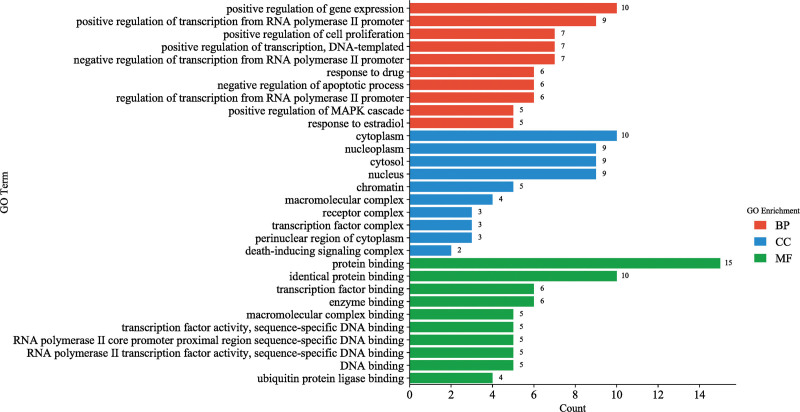
GO enrichment analysis of key targets. Note: red represents biological processes, blue represents cellular component, and green represents molecular function. GO = gene ontology.

The targets were imported into the DAVID data platform for GO annotation analysis using “homo sapiens” as the study context. A total of 210 entries were obtained for GO enrichment analysis, including 159 entries for biological process (BP), 12 entries for cell composition (CC), and 39 entries for molecular function (MF). The top 10 entries were visualized (Q < 0.05) and ranked by gene size. BP was mainly involved in the positive regulation of gene expression, positive regulation of transcription from RNA polymerase II promoter, positive regulation of cell proliferation, positive regulation of transcription, DNA-templated, negative regulation of transcription from RNA polymerase II promoter, response to drug, negative regulation of apoptotic process, regulation of transcription from RNA polymerase II promoter, positive regulation of mitogen-activated protein kinase (MAPK) cascade, and response to estradiol. CC was mainly involved in cytoplasm, nucleoplasm, cytosol, nucleus, chromatin, macromolecular complex, receptor complex, transcription factor complex, perinuclear region of cytoplasm, and death-inducing signaling complex. MF was mainly involved in protein binding, identical protein binding, transcription factor binding, enzyme binding, macromolecular complex binding, transcription factor activity, sequence-specific DNA binding, RNA polymerase II core promoter proximal region sequence-specific DNA binding, RNA polymerase II transcription factor activity, sequence-specific DNA binding, DNA binding, and ubiquitin protein ligase binding. All 3 processes with respective involvement are shown in Figure 4.

**Figure 5.**
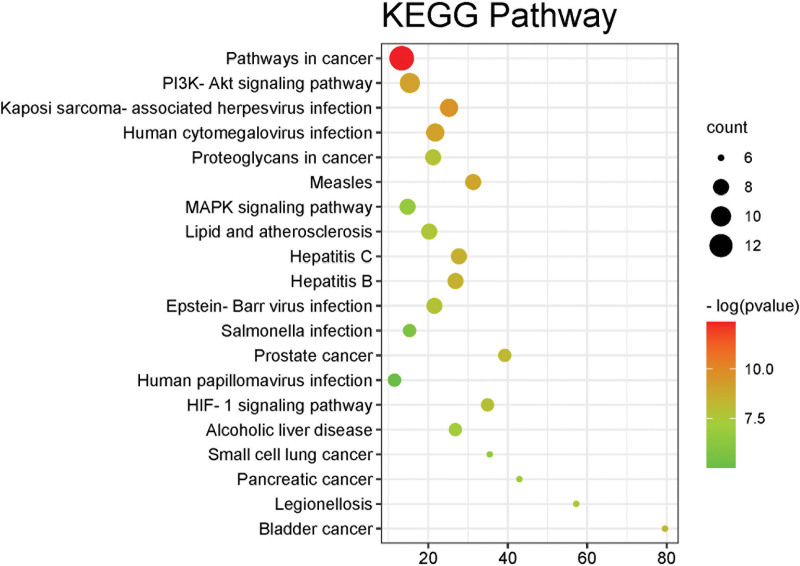
KEGG enrichment analysis of key targets Note: the x-axis represents the gene ratio, the y-axis represents the enriched pathways, the size of the dots indicates the gene number, and the color of the dots represents the *P* value. KEGG = Kyoto encyclopedia of genes and genomes.

98 pathways were obtained by KEGG enrichment analysis through the DAVID data platform, and the top 20 pathways were screened for visualization, which showed that the key targets were mainly involved in Pathways in cancer, phosphatidylinositol-3-kinaseRAC–serine/threonine-protein kinase signaling pathway, Kaposi sarcoma-associated herpesvirus infection, Human cytomegalovirus infection, Measles, Hepatitis C, Hepatitis B, Epstein-Barr virus infection, Proteoglycans in cancer, Lipid and atherosclerosis, MAPK signaling pathway, Prostate cancer, aryl hydrocarbon receptor nuclear translocator signaling pathway, Alcoholic liver disease, Salmonella infection, Human papillomavirus infection, Bladder cancer, Legionellosis, Pancreatic cancer, Small cell lung cancer as shown in Figure 5 and Table 5.

**Table 5:** Signaling pathways.

ID	Signaling pathway	Gene number
hsa05200	Pathways in cancer	13
hsa04151	PI3K-Akt signaling pathway	10
hsa05167	Kaposi sarcoma-associated herpesvirus infection	9
hsa05163	Human cytomegalovirus infection	9
hsa05162	Measles	8
hsa05160	Hepatitis C	8
hsa05161	Hepatitis B	8
hsa05169	Epstein-Barr virus infection	8
hsa05205	Proteoglycans in cancer	8
hsa05417	Lipid and atherosclerosis	8
hsa04010	MAPK signaling pathway	8
hsa05215	Prostate cancer	7
hsa04066	HIF-1 signaling pathway	7
hsa04936	Alcoholic liver disease	7
hsa05132	Salmonella infection	7
hsa05165	Human papillomavirus infection	7
hsa05219	Bladder cancer	6
hsa05134	Legionellosis	6
hsa05212	Pancreatic cancer	6
hsa05222	Small cell lung cancer	6

HIF-1 = aryl hydrocarbon receptor nuclear translocator, MAPK = mitogen-activated protein kinase, PI3K-Akt = phosphatidylinositol-3-kinaseRAC–serine/threonine-protein kinase.

### 3.6. Results of molecular docking

**Figure 6.**
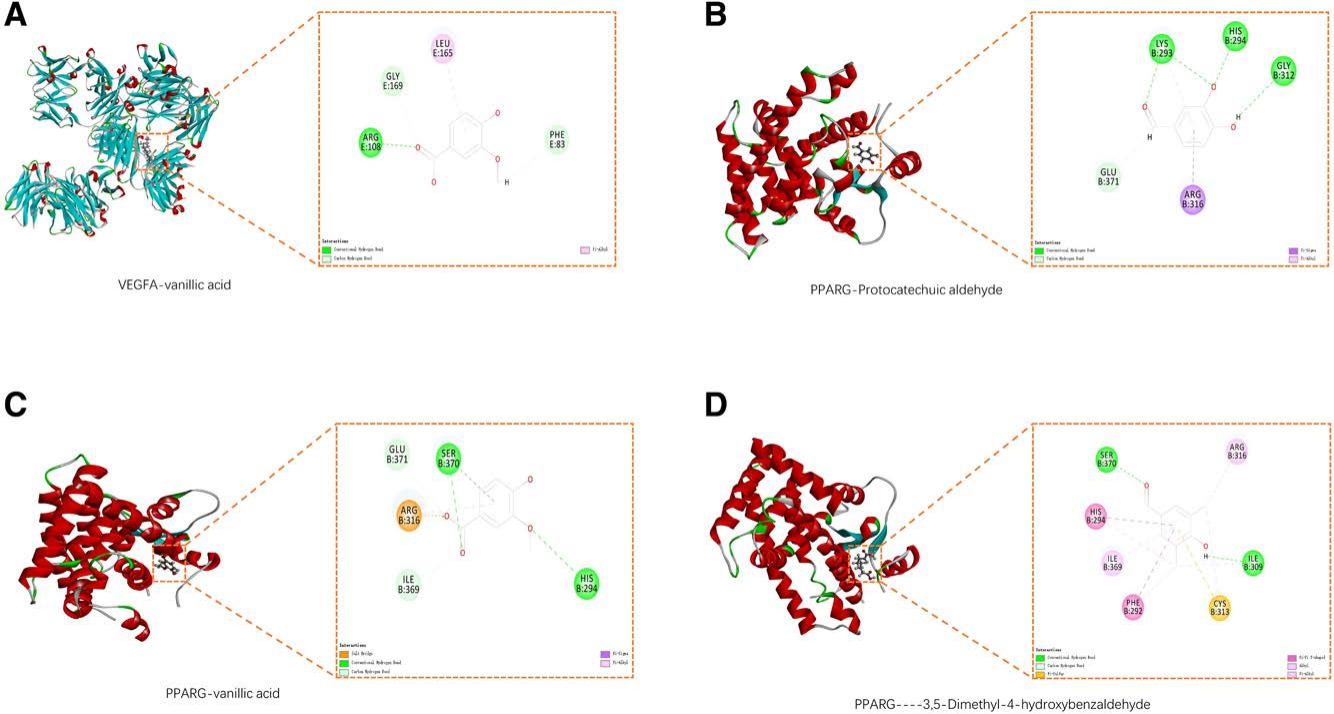
Core compound-core target molecular docking diagram. (A) Molecular docking diagram of VEGFA and Vanillic acid; (B) molecular docking diagram of PPARG and Protocatechuic aldehyde; (C) molecular docking diagram of PPARG and Vanillic acid; (D) molecular docking diagram of PPARG and 3,5-dimethyl-4-hydroxybenzaldehyde.

TP53, INS, IL6, VEGFA, MYC, CASP3, ESR1, EGF, CCND1, PPARG, ERBB2, NFKBIA, TLR4, RELA, and CASP8 were selected to find the corresponding effective chemical components for molecular docking, and the docking scores were used to determine the affinity. The docking results are shown in Figure 6 and Table 6. It can be seen that the binding scores between PPARG and VEGFA and 3,5-dimethyl-4-hydroxybenzaldehyde, protocatechuic-aldehyde, and vanillic acid were high and had a good affinity.

**Table 6:** Results of molecular docking.

Core target	Component	Docking scores
PPARG	3,5-dimethyl-4-hydroxybenzaldehyde	78.82
PPARG	Protocatechuic aldehyde	71.40
PPARG	Vanillic acid	74.71
VEGFA	Vanillic acid	140.19

### 3.7. Comparison of clinical efficacy between the 2 groups

After treatment, the total effective rate of the control and treatment groups was 87.69% and 96.92%, respectively, and the difference between the 2 groups was statistically significant (*P* < .05) in Table 7.

**Table 7:** Comparison of clinical efficacy.

Group	No. of cases	Clinical control	Apparent effect	Effective	Ineffective	Total effective rate
Treatment	49	5	22	20	2	95.9%*
Control	50	10	15	18	7	86.0%

* *P *< .05 versus control group after treatment.

### 3.8. Comparison of the main symptoms and signs between the 2 groups

**Figure 7.**
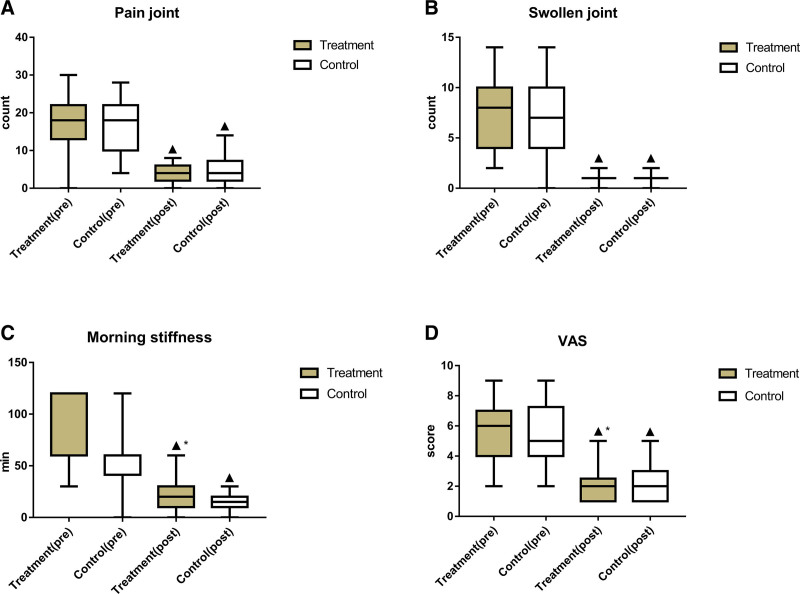
Comparison of the main symptoms and signs between the 2 groups. (A) Comparison of pain joint count between the 2 groups; (B) comparison of swollen joint count between the 2 groups; (C) comparison of morning stiffness (min) between the 2 groups; (D) comparison of VAS between the 2 groups. Bars represent Mean ± SD (n = 15). ^▲^*P* < .05 vs same group before treatment; ^*^P < .05 vs control group after treatment. VAS = visual analog scale.

After treatment, the time of morning stiffness, swollen joint count, joint pain count, and VAS score were significantly reduced in both groups compared with those before treatment. The improvement in these indexes was significantly bigger in the treatment group than in the control group (*P* < .05), as shown in Figure 7 and Table 8.

**Table 8:** Comparison of the main symptoms and signs between the 2 groups (‾*X* ± S).

Group	No. of cases	Timing of observation	Pain joint count	Swollen joint count	Morning stiffness (min)	VAS
Treatment	49	Pre-treatment	17.39 ± 7.05	6.86 ± 3.44	70.82 ± 31.21	5.53 ± 1.86
		Post-treatment	4.10 ± 2.30▲	0.94 ± 0.55▲	15.04 ± 7.27▲*	1.82 ± 0.95▲*
Control	50	Pre-treatment	16.72 ± 6.81	6.80 ± 3.18	63.90 ± 34.05	5.36 ± 2.19
		Post-treatment	4.88 ± 3.47▲	0.90 ± 0.41▲	25.82 ± 17.88▲	2.30 ± 1.10▲

VAS = visual analog scale.

▲ *P* < .05 versus same group before treatment;

* *P *< .05 versus control group after treatment.

### 3.9. Comparison of ESR, CRP, RF, and anti-CCP levels between the 2 groups

**Figure 8.**
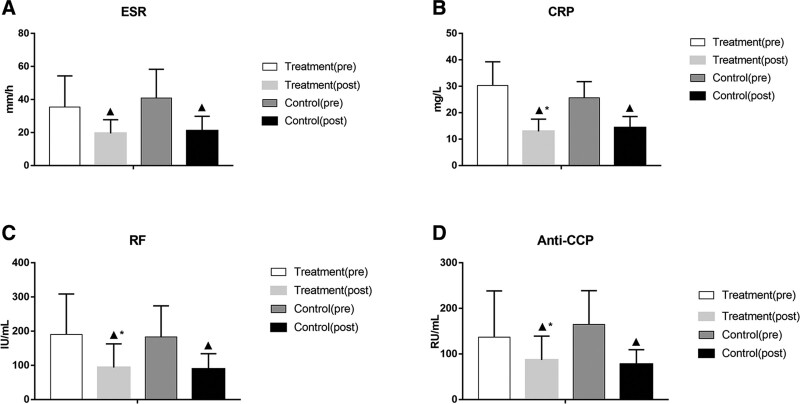
Comparison of ESR, CRP, RF, and anti-CCP levels between the 2 groups. (A) Comparison of ESR levels between the 2 groups; (B) comparison of CRP levels between the 2 groups; (C) comparison of RF levels between the 2 groups; (D) comparison of anti-CCP levels between the 2 groups. Bars represent Mean ± SD (n = 15). ^▲^*P* < .05 vs same group before treatment; ^*^P < .05 vs control group after treatment. anti-CCP = anti-cyclic citrullinated peptide. CRP = C-reactive protein, ESR = erythrocyte sedimentation rate, RF = rheumatoid factor.

After treatment, the levels of ESR, CRP, RF, and anti-CCP in both groups were significantly lower than those before treatment (*P* < .05). The levels these indexes in the treatment group were significantly lower than those in the control group (*P* < .05), as shown in Figure 8 and Table 9.

**Table 9:** Comparison of ESR, CRP, RF, and anti-CCP levels between the 2 groups.

Group	No. of cases	Timing of observation	ESR (mm·h^−1^)	CRP (mg·L^−1^)	RF (IU·mL^−1^)	Anti-CCP s (RU·mL^−1^)
Treatment	49	Pre-treatment	40.80 ± 21.06	25.61 ± 14.26	182.76 ± 89.13	164.69 ± 77.11
		Post-treatment	17.49 ± 8.77▲	6.15 ± 4.30▲*	91.40 ± 45.02▲*	74.16 ± 32.23▲*
Control	50	Pre-treatment	35.38 ± 19.5	30.34 ± 12.93	190.32 ± 93.82	136.52 ± 86.90
		Post-treatment	18.92 ± 8.25▲	8.93 ± 4.65▲	118.67 ± 69.09▲	101.84 ± 52.27▲

anti-CCP = anti-cyclic citrullinated peptide, CRP = C-reactive protein, ESR = erythrocyte sedimentation rate, RF = rheumatoid factor.

▲ *P* < .05 versus same group before treatment;

* *P* < .05 versus control group after treatment.

### 3.10. Comparison of TNF-α, IL-6, and IL-17 levels in the 2 groups

**Figure 9.**
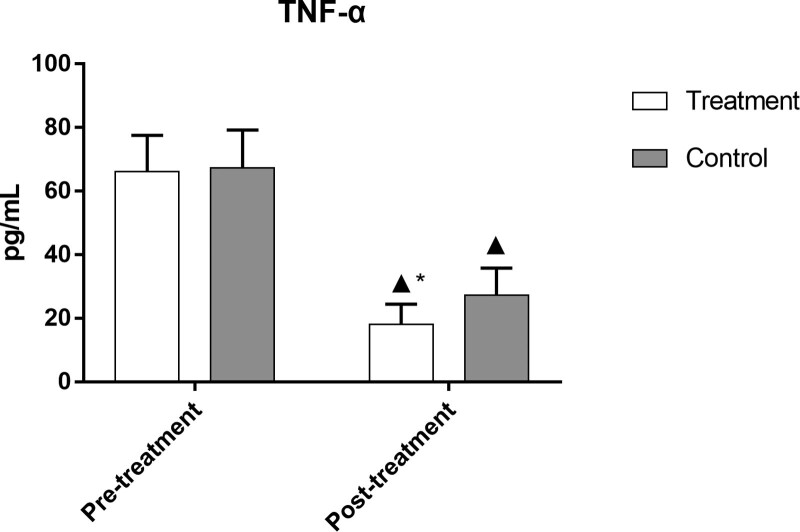
Comparison of TNF-α levels in the 2 groups. Bars represent Mean ± SD (n = 15). ^▲^*P* < .05 vs same group before treatment; ^*^P < .05 vs control group after treatment. TNF-α = tumor necrosis factor.

**Figure 10.**
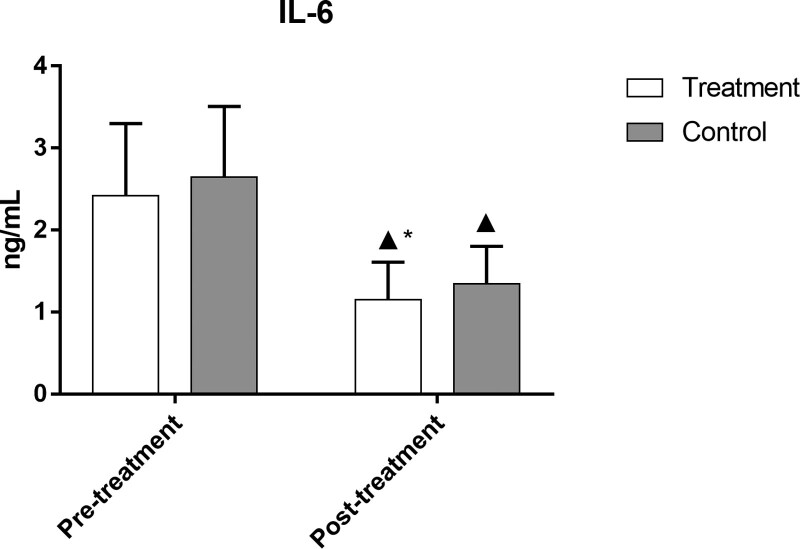
Comparison of IL-6 levels in the 2 groups. Bars represent Mean ± SD (n = 15). ^▲^*P* < .05 vs same group before treatment; ^*^P < .05 vs control group after treatment. IL-6 = interleukin-6.

**Figure 11.**
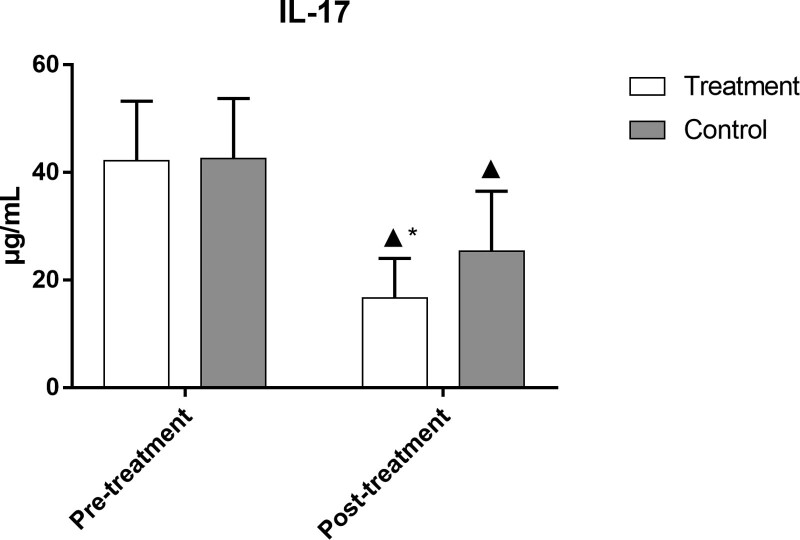
Comparison of IL-17 levels in the 2 groups. Bars represent Mean ± SD (n = 15). ^▲^*P* < .05 vs same group before treatment; ^*^P < .05 vs control group after treatment. IL-17 = interleukin-17.

After treatment, the levels of TNF-α, IL-6, and IL-17 in both groups were significantly lower than those before treatment (*P *< .05). The levels of these indicators in the treatment group were significantly lower than those in the control group (*P *< .05), as demonstrated in Figures 9–11 and Table 10.

**Table 10:** Comparison of TNF-α, IL-6, and IL-17 levels in the 2 groups.

Group	No. of cases	Timing of observation	TNF-α (pg·mL^−1^)	IL-6 (ng·L^−1^)	IL-17 s (µg·L^−1^)
Treatment	49	Pre-treatment	66.17 ± 11.39	2.42 ± 0.88	42.16 ± 11.13
		Post-treatment	18.03 ± 6.46▲*	1.15 ± 0.46▲*	16.66 ± 7.36▲*
Control	50	Pre-treatment	67.30 ± 11.90	2.65 ± 0.86	42.60 ± 11.16
		Post-treatment	27.28 ± 8.48▲	1.34 ± 0.46▲	25.31 ± 11.22▲

IL-17 = interleukin-17, IL-6 = interleukin-6, TNF-α = tumor necrosis factor.

▲ *P* < .05 versus same group before treatment;

* *P* < .05 versus control group after treatment.

### 3.11. Comparison of immune function between the 2 groups

**Figure 12.**
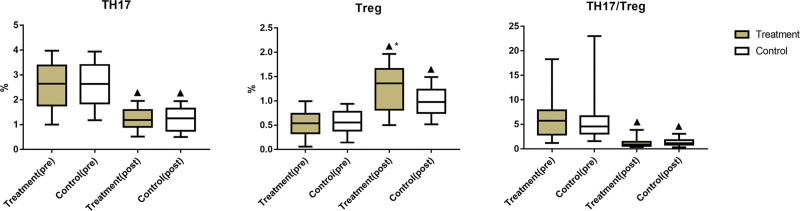
Comparison of immune function between the 2 groups. Comparison of Th17/%, Treg/%, Th17/Treg between the 2 groups. Bars represent Mean ± SD (n = 15). ^▲^*P* < .05 vs same group before treatment; ^*^P < .05 vs control group after treatment. Th17 = helper T cells 17, Treg = regulatory T cells.

After treatment, the Th17 ratio and Th17/Treg value decreased in both groups, while the Treg cell ratio increased in both groups (*P* < .05). The improvement in these indexes was significantly bigger in the treatment group (*P* < .05), as shown in Figure 12 and Table 11.

**Table 11:** Comparison of immune function between the 2 groups.

Group	No. of cases	Timing of observation	Th17/%	Treg/%	Th17/Treg
Treatment	49	Pre-treatment	2.53 ± 0.88	0.54 ± 0.23	5.93 ± 3.41
		Post-treatment	1.23 ± 0.41▲	1.26 ± 0.46▲*	1.18 ± 0.73▲
Control	50	Pre-treatment	2.62 ± 0.84	0.57 ± 0.22	5.61 ± 3.86
		Post-treatment	1.21 ± 0.46▲	0.99 ± 0.29▲	1.34 ± 0.67▲

Th17 = helper T cells 17, Treg = regulatory T cells.

▲ *P* < .05 versus same group before treatment;

* *P* < .05 versus control group after treatment.

### 3.12. Comparison of adverse reactions between the 2 groups

There were no adverse events that seriously affected the treatment of patients in both groups. Two patients had a mild elevation of glutamate transaminase, 1 patient had nausea, and 1 patient had thrombocytopenia during the treatment in the treatment group, and the incidence of adverse reactions was 8.2%. In the control group, 2 patients had a mild elevation of glutamate transaminase, and 1 patient had vomiting during the treatment period, with an adverse reaction incidence of 6.0%. All of them received symptomatic treatment, which did not affect RA treatment, and there was no significant difference in the incidence of adverse reactions between the 2 groups.

## 4. Discussion

The clinical drugs used for the treatment of RA can be broadly classified into 2 categories according to their mode of action: those for the improvement of symptoms and those for the improvement of cartilage metabolism. However, symptom improvement drugs cannot fundamentally reverse the development of RA pathology. With the development of Chinese medicine extraction technology, the application of Chinese medicine preparation extracts for the treatment of RA has been increasingly reported and supported by relevant basic experimental studies. JSQS can effectively protect chondrocytes and maintain the integrity of cartilage, reduce the level of inflammatory cytokines, and play a certain therapeutic role in RA.^[10]^ In this study, we elaborated the material basis and molecular mechanism of RA treatment with JSQS through network pharmacology and verified the effect through clinical control trials. It was confirmed that JSQS has multicomponent, multipathway, and multitarget synergistic intervention characteristics in RA and can effectively reverse the pathological development of RA and improve the function of the knee joint and quality of life.

In this study, 141 chiropteran capsule targets were mapped to 4791 RA target genes, 118 anti-RA key targets of JSQS were obtained, and a JSQS-key target-RA composite target network map was constructed. The quercetin in *Panax notoginseng*, vanillic acid, scopoletin, physcion in *Zaocys dhumnades*, 3,5-dimethyl-4-hydroxybenzaldehyde in *Cibotium barometz*, kaempferol in *Paeonia lactiflora*, and protocatechuic in *Gardneria angustifolia* Bioinformatics were the main active chemical components in the composite target network. The network showed that 1 active chemical component could correspond to 1 or even multiple targets, and multiple targets corresponded to the same active chemical component, indicating that the JSQS has the characteristics of multicomponent and multitarget synergistic intervention in RA treatment.

Quercetin has an anti-inflammatory effect on RA by reducing the ratio of MMP-13/TIMP-1, promoting the inhibition of cartilage extracellular matrix degradation, and protecting articular cartilage.^[10]^ Scopoletin is a coumarin-like compound with anti-swelling, anti-inflammatory, analgesic, antibacterial, and acaricidal biological activities.^[11]^ Physcion, as its mechanism of action on TNF-α-induced RA was identified by immunofluorescence, western blot, and reverse-transcription PCR, dose-dependently inhibits the expression of ERK1/2 and p38MAPK in fibroblast-like synovial cells to attenuate the inflammatory response.^[12]^ In terms of apoptosis induction,^[13]^ physcion at concentrations of 6 to 10 mol/L significantly inhibits the activity of synovial cells in a mixture of type II collagen and incomplete Freund adjuvant to induce RA model rats. Pan Shuhan et al^[14]^ confirmed that physcion (40 mg/kg) significantly reduces the expression of Cat-G, Cat-S, and autophagy marker LC3 in synovial tissues using immunohistochemistry and enzyme-linked immunosorbent assay in an equal volume emulsion of collagen-induced arthritis rat model. Physcion has been found to present a quantitative and temporal relationship in inhibiting the metastasis and proliferation of RA–fibroblast-like synoviocytes cultured in vitro.^[15]^ At the cellular level, kaempferol exerts anti-inflammatory effects, reducing the production and mRNA expression of pro-inflammatory cytokines, such as thymic stromal lymphopoietin, TNF-α, and interleukin-18.^[16]^

The results of KEGG enrichment analysis showed that JSQS were mainly used to treat RA through the pathways in cancer, phosphatidylinositol-3-kinaseRAC–serine/threonine-protein kinase signaling pathway, MAPK signaling pathway, and aryl hydrocarbon receptor nuclear translocator signaling pathway, among others. The topological analysis of this study showed that TP53, INS, IL6, VEGFA, MYC, CASP3, ESR1, EGF, CCND1, PPARG, ERBB2, NFKBIA, TLR4, RELA, and CASP8 are the core key targets. Molecular docking was performed according to the corresponding active chemical components, suggesting binding fractions of >70 between PPARG and VEGFA and 3,5-dimethyl-4-hydroxybenzaldehyde, protocatechuic-aldehyde, vanillic acid, which had good binding energy. It indicated that these active chemical components might play an important role in RA treatment. However, further in vivo and in vitro studies are needed to confirm the possible existence of a therapeutic axis.

PPAR, a member of the nuclear receptor superfamily that regulates lipid, glucose, and amino acid metabolism,^[17]^ belongs to the nuclear hormone receptors and consists of 3 different isoforms, namely PPARα, PPARγ (PPARG), and PPARβ/δ. PPARγ activators or compounds that positively regulate PPAR gene expression may represent a new class of nonsteroidal anti-inflammatory drugs for local or systemic treatment of many inflammatory conditions. PPAR activation has been reported to reduce the level of Th2 immune response,^[18]^ and high expression of PPARG inhibits IL-6 and TNF-α by suppressing nuclear factor kappa B activity, which, in turn, suppresses the overexcited inflammatory response. Additionally, PPARG activates osteoclasts, which cause bone destruction in RA patients, preserving the patient joint structure. The active osteoclasts cause bone destruction in RA patients, resulting in abnormalities in joint structure, resulting in morning stiffness, reduced motion, and long-term joint deformation.

Synovitis and vasculitis of joints are the basic pathological factors of RA. Neovascularization is considered to be one of the main factors in the formation and maintenance of pannus in RA, and some studies have shown that VEGF can increase vascular permeability and play a very critical role in the formation and development of pannus in RA synovitis.^[19,20]^ Furthermore, it is important in promoting the formation and development of inflammation, especially for the formation of chronic inflammation.

This study identified the possible material basis and molecular mechanism of JSQS in RA treatment. Thus, our team conducted a controlled clinical trial to verify the therapeutic effect of JSQS in RA treatment. The results showed that the capsules could effectively improve morning stiffness, joint pain, VAS score, other indicators of RA, clinical symptoms, and patient quality of life. The results of laboratory tests, such as ESR, CRP, RF, and anti-CCP, also indicated effective disease control, reduced levels of inflammation-related factors, such as TNF-α, IL-6, and IL-17, and significantly improved Th17/Treg balance. In conclusion, the multicomponent, multipathway, and multitarget synergistic interventions of JSQS can significantly improve clinical symptoms and quality of life of RA patients and delay the progression of the disease. Insufficient sample size is a regret of this study due to time and financial constraints. After obtaining the preliminary results, we will carry out a multicenter study, and at the same time, we will optimize the design of the study protocol on the line, in order to provide a theoretical basis and guidance for the clinical application of Chinese medicine.

## Author contributions

**Data curation:** Nannan Zhang.

**Formal analysis:** Xin Peng.

**Funding acquisition:** Xueming Yao.

**Project administration:** Cong Huang.

**Software:** Nannan Zhang.

**Supervision:** Wukai Ma, Xueming Yao.

**Writing – original draft:** Yujie Li, Xin Peng.

**Writing – review & editing:** Yuanxing Qin, Xudong Zhang.
